# Endocytic trafficking induces lateral root founder cell specification in *Arabidopsis thaliana* in a process distinct from the auxin-induced pathway

**DOI:** 10.3389/fpls.2022.1060021

**Published:** 2023-01-16

**Authors:** Stefanía Morales-Herrera, Carlos Rubilar-Hernández, Patricio Pérez-Henríquez, Lorena Norambuena

**Affiliations:** Plant Molecular Biology Centre, Department of Biology, Facultad de Ciencias, Universidad de Chile, Santiago, Chile

**Keywords:** endocytic trafficking, founder cell specification, lateral root (LR) formation, organogenesis, Sortin2

## Abstract

Plants can modify their body structure, such as their root architecture, post-embryonically. For example, *Arabidopsis thaliana* can develop lateral roots as part of an endogenous program or in response to biotic and abiotic stimuli. Root pericycle cells are specified to become lateral root founder cells, initiating lateral root organogenesis. We used the endocytic trafficking inducer Sortin2 to examine the role of endomembrane trafficking in lateral root founder cell specification. Our results indicate that Sortin2 stimulation turns on a *de novo* program of lateral root primordium formation that is distinct from the endogenous program driven by auxin. In this distinctive mechanism, extracellular calcium uptake and endocytic trafficking toward the vacuole are required for lateral root founder cell specification upstream of the auxin module led by AUX/IAA28. The auxin-dependent TIR1/AFB F-boxes and auxin polar transport are dispensable for the endocytic trafficking–dependent lateral root founder cell specification; however, a different set of F-box proteins and a functional SCF complex are required. The endocytic trafficking could constitute a convenient strategy for organogenesis in response to environmental conditions.

## Introduction

Plants perceive biotic and abiotic stimuli and respond to changing environmental conditions. Post-embryonic plant development provides exceptional flexibility for adaptation to environmental changes and adverse conditions. For instance, plant root growth is modified in response to nutrient and water availability, biotic and abiotic stresses, and interactions with the soil microbiome (Nibau et al., 2008; Lima et al., 2010; Miura et al., 2011; Gruber et al., 2013; Giehl and von Wirén, 2014; Pieterse et al., 2014). Root shape and spatial disposition within the soil determines the root system architecture (RSA). During a plant’s lifespan, the RSA is plastically modified by adjusting the type, number, size, and arrangement angle of root organs to explore the soil environment (Gruber et al., 2013; Giehl and von Wirén, 2014; Rogers and Benfey, 2015).

In species such as Arabidopsis (*Arabidopsis thaliana*), the RSA is highly modified by the development of lateral roots (LRs). In Arabidopsis and most other dicots, LRs originate from the pericycle cell layer (Casimiro et al., 2001). During LR priming, two adjacent pericycle cells located at the basal root meristem become LR founder cells (LRFCs) (De Smet et al., 2007; Van Norman et al., 2013). These cells divide asymmetrically during LR initiation (LRI) (Dubrovsky et al., 2000; Peret et al., 2009). After LRI, periclinal cell divisions result in a two-cell layered LR primordium (LRP), and consecutive divisions create a dome-shaped primordium that emerges from the main root (Malamy and Benfey, 1997; De Smet et al., 2007). Iterative production of LRs in plants follows a regular pattern, suggesting tight control of LRFC determination (Moreno-Risueno et al., 2010). This regular pattern is controlled by periodic oscillation of gene expression at the root basal meristem, which is the first step of LR formation and is known as LR pre-patterning (Moreno-Risueno et al., 2010; Van Norman et al., 2013). LR development can also be modified depending on the surrounding environment. The sequential steps during the oscillation, LRFC specification and LR formation are controlled by endogenous and exogenous signals to determine both the positioning and development of LRP formation and, consequently, the RSA.

The molecular mechanisms driving LR priming have not yet been described. LRFCs perceive and respond to an activation signal, initiating LRI (Van Norman et al., 2013). Activation of the transcription factor GATA23 is important in controlling LRFC identity (De Rybel et al., 2010). Moreover, accumulation of the membrane-associated kinase regulator MAKR4 is required to specify LRFCs and/or activate LRI (Xuan et al., 2015). These two molecular events are proposed to be upstream of morphological changes that take place just before LRI, allowing the first division of the LRFC (De Rybel et al., 2010; Vermeer et al., 2014).

Auxin is the key hormone for inducing and regulating LR formation. Auxin binds to TRANSPORT INHIBITOR RESPONSE1 (TIR1)/AUXIN-SIGNALING F-BOX1-5 (TIR1/AFBs) proteins, leading to the activation of the auxin receptor complex SCF^TIR1/AFBs^ with E3 ubiquitin ligase activity (Dharmasiri et al., 2003; Parry et al., 2009; Salehin et al., 2015). SCF^TIR1/AFBs^ is required for LR formation and also responds to exogenous auxin (Dharmasiri et al., 2005; Xuan et al., 2015). Auxin maxima are required for LRFC nuclear migration and trigger the transcriptional response required for LRI (Vanneste et al., 2005; De Rybel et al., 2010). Although the importance of auxin in LR formation has been thoroughly documented, its particular role in LRFC specification has not been elucidated (Overvoorde et al., 2010; Van Norman et al., 2013). Periodic oscillations in auxin responses along primary roots coincide with the location of new LR development, known as the prebranching sites (Moreno-Risueno et al., 2010). Genes with expression patterns that oscillate in phase or in antiphase with auxin response reporters have been identified (Moreno-Risueno et al., 2010). However, alterations in auxin level or signaling seem insufficient to alter the oscillatory system, suggesting that auxin maxima are not necessarily the signal that triggers LRFC priming (Moreno-Risueno et al., 2010; Van Norman et al., 2013). Mechanical stimulus and bacterial quorum-sensing molecules trigger LR formation in what has been claimed to be an auxin-independent mechanism (Ditengou et al., 2008; Ortiz-Castro et al., 2008; Richter et al., 2009). Therefore, it is possible that other molecular mechanisms can determine the identity of LRFC.

The synthetic chemical Sortin2 induces endocytosis from the plasma membrane (PM) toward the vacuole in Arabidopsis (Zouhar et al., 2004; Pérez-Henríquez et al., 2012). Using this chemical tool, we reported a mechanism for LR formation dependent on endomembrane trafficking. Specifically, Sortin2 increases the number of emerged LRs through a process that is abolished by blocking endocytic protein trafficking toward the vacuole (Pérez-Henríquez et al., 2012). This evidence suggested the existence in Arabidopsis of a molecular developmental pathway dependent on protein trafficking. In this case, Sortin2 is an agonist of the pathway, resulting in the formation of new LRs in Arabidopsis. Here, we show that the endocytic trafficking induction by Sortin2 stimulates *de novo* LR priming. Endocytic trafficking–dependent LRFC differentiation occurs upstream of the auxin-regulated mechanism; however, auxin signaling is required for further LR development. The agonist Sortin2 promoted a mechanism that requires extracellular Ca^2+^, which is distinctive to auxin-triggered LR formation.

## Materials and methods

*Arabidopsis thaliana* seeds were sown and grown in 1% sucrose Murashige & Skoog solid (MSS) and/or liquid (MSL) medium as described by Pérez-Henríquez et al., 2012. Seedlings were grown vertically at 22°C with a 16-hour-light/8-hour-dark photoperiod in a culture chamber. The wild-type, mutant, and reporter lines were requested from laboratories abroad. Mutant phenotypes and reporter patterns were confirmed in our experimental conditions.

### Chemical treatments

Experiments were performed with 7-day-old seedlings in MSS that had previously been exposed to 6200 lumens of light in the photoperiod and growing conditions described above for 3 days. Seedlings were challenged with 25 μg mL^−1^ Sortin2 dissolved in dimethyl sulfoxide (DMSO). Control treatments received equivalent amounts of DMSO. An LR-inducible system (LRIS) was established by sowing seeds in MSS containing 10 μM *N*-1-naphthylphthalamic acid (NPA) dissolved in DMSO. After 7 days, seedlings were transferred to NPA-free MSS and subjected to different treatments. For the treatments, 1-naphthaleneacetic acid (NAA), auxinole, and wortmannin were dissolved in DMSO.

Segmented agar plates (SAPs), as described previously (Zhang and Forde, 1998), allowed local application of different treatments on a particular region of seedlings. The sections of SAPs were separated by a layer of air (2 mm) to avoid chemical diffusion through growth medium. Seedlings were transferred to SAPs, placing the cotyledons, hypocotyl, and the first section of the main root on section I and the apical root tip on section III. After the treatment, developed LRP and LR events over each section of SAP were quantified.

### LR architecture analysis

After treatments, seedlings were fixed with 70% ethanol for 24 hours. Roots were cleared with 90% lactic acid for at least 24 hours. Microscopy was used to determine the number of LRPs and emerged LRs, which were normalized to the primary root length to obtain the index of each of them. For scoring the LRP stage, roots were cleared as described previously (Malamy and Benfey, 1997). GUS staining of the reporter p*CYCB1;1*::GUS was evaluated as described by Norambuena et al. (2009). The GUS positive events were quantified manually under the light microscope. The GUS spots within primary root were considered LRP.

### Confocal microscopy

Imaging was performed with a Zeiss LSM 710 confocal microscope. For PIN1-GFP images, GFP was excited using a 488-nm laser and detected with a 490–598-nm emission filter. Cyan fluorescent protein (ECFP) and cpVenus were excited with a 458-nm line of an argon laser, and their emission was captured from 465–500 nm and 520–570 nm, respectively. The image dimension was 1024 × 1024, and the line average was 4. Images were processed with Zen 2012 Blue edition or FIJI.

### Calcium sensor imaging

Calcium content was evaluated with the calcium reporter line yellow cameleon using the Arabidopsis line NES-YC3.6 (Krebs et al., 2012). The ECFP and cpVenus signals were detected by confocal microscopy by simultaneously taking images every 12 seconds over 8 minutes of the experiment. Five-day-old NES-YC3.6 seedlings were placed in a custom-built perfusion chamber containing MSL medium as described by Krebs et al. (2012). Seedlings were treated with MSL containing 1% DMSO for 180 seconds. Then, the solution was replaced with MSL containing 50 μg/mL Sortin2 or 1% DMSO (control). The observation proceeded for another 300 seconds. The ECFP and cpVenus signals at each time were quantified, and the background signal was subtracted. The cpVenus/ECFP ratio was calculated and normalized by the ratio value at the beginning of the experiment (time zero) as described by Krebs et al. (2012).

### RNA isolation and transcript level quantification

RNA was extracted from seedlings using an InviTrap Spin Plant RNA Mini Kit (Invitek). cDNA was generated using Impron (Promega) with 1 µg of purified RNA. Transcript levels were measured by RT-qPCR in a MX3000P qPCR System using Brilliant III ultra-Fast qPCR Master Mix. Primers for amplifying *GATA23* (forward 5′-CGGACGAACTCTTCTACAAAGG-3′ and reverse 5′-ATTCGTCGTCGAAGGTGTAATC-3′) and *MAKR4* (forward 5′-AGACGATCAGAGTTATTGGGTATTC-3′ and reverse 5′-CCTCCTTTAGACTCCTTCGTTTC-3′) were designed. Transcript levels were normalized to the invariable transcript level of *AP2M*. Relative expression was calculated using the 2^−ΔΔCT^ method (Pfaffl, 2001).

### Data and statistical analysis

All graphs show means and standard errors. Sample sizes and statistical methods used are included in each figure legend. The statistical significance was calculated by analysis of variance and by Student’s *t*-test. All analyses were performed with GraphPad Prism 9.3.1 (GraphPad, San Diego, CA, USA).

## Results

### Sortin2 induces endocytic trafficking and division of pericycle cells

The ability of Sortin2 to trigger LR formation is linked to its effect on endocytic trafficking in epidermal root cells (Pérez-Henríquez et al., 2012). Perez-Henríquez et al. showed that PM-endosome-localized proteins, such as BRI1 and PIN2, are driven to the vacuole due to the Sortin2 bioactivity. However, whether Sortin2 affects the trafficking to the vacuole in the pericycle cell layer was not determined.

**Figure 1:**
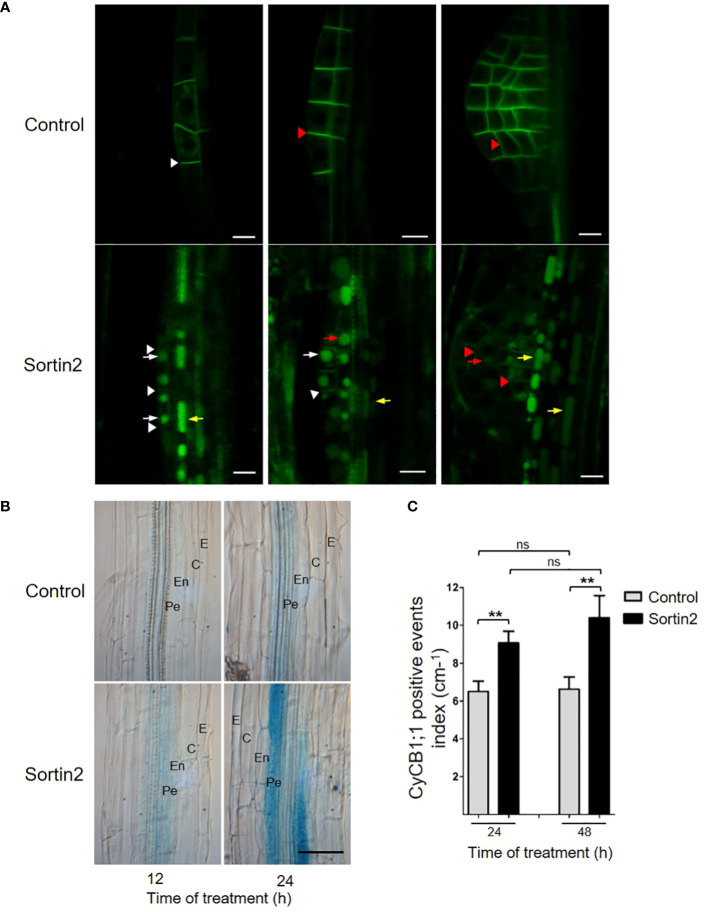
Sortin2 exerts its effect in pericycle cells. **(A)** *pPIN1::PIN1-GFP* transgenic Arabidopsis seedlings were treated for 12 hours with Sortin or control conditions. The localization of PIN1-GFP (green) in roots was evaluated. Stages of lateral root primordium were defined according to Malamy and Benfey (1997). Images are representative of seedlings in three independent experiments (*n* ≥ 9 seedlings). Arrows and arrowheads indicate the plasma membrane and vacuoles, respectively, in pericycle cells (yellow), lateral root founder cells (white), and primordium cells (red). Scale bars = 10 μm. **(B, C)** Seven-day-old p*CYCB1;1*::GUS reporter line seedlings were treated with Sortin2 for 12, 24, and 48 hours (or not treated, for controls), and their GUS activity was evaluated. **(B)** Brightfield images of GUS activity in seedlings treated with Sortin2 for 12 and 24 hours, with the following root cell layers indicated: epidermis (E), cortex **(C)**, endodermis (En), and pericycle (Pe). Scale bar = 50 µm. **(C)** p*CYCB1;1*::GUS-positive events within the main root were manually quantified under the light microscope in Sortin2-treated (black bar) and control (gray bar) conditions. The results from five experimental replicates (*n* ≥ 26 seedlings) were analyzed using Student’s *t*-tests; brackets denote statistical differences between conditions (**p < 0.01; ns, not significant).

To test Sortin2 bioactivity in pericycle cells, we analyzed the behavior of the PM-endosome-localized PIN1 protein, since *PIN2* is not expressed in this cell layer (Kleine-Vehn and Friml, 2008). We tested the effect of Sortin2 on the trafficking of a PIN1-GFP fusion protein using the transgenic line expressing *pPIN1::PIN1-GFP* (Friml et al., 2003). Indeed, Sortin2 stimulated the accumulation of PIN1-GFP in the vacuole in pericycle cells (**Figure 1A**). The effect of Sortin2 was also visualized in LRFCs as well in cells of the primordia at different stages of development (**Figure 1A**). The bioactive compound also triggered PIN1:GFP trafficking in other cell layers of the differentiation zone of the primary root (**Figure 1A**). This result suggests that Sortin2 induced an acceleration of endocytic trafficking toward the vacuole in root cell layers deeper than the endodermis. More importantly, endocytic trafficking proceeded in the cell layer where LRFC differentiation occurs.

Sortin2 promotes the activation of the mitotic promoter pCYC1;1, which is associated with LR formation (Beeckman et al., 2001), indicating that it promotes cell division events leading to LRP formation (Pérez-Henríquez et al., 2012). To determine the spatiotemporal effect of Sortin2 along root cell layers, we evaluated the induction of the reporter line p*CYCB1::GUS* (Ferreira et al., 1994a; Ferreira et al., 1994b) by Sortin2. After 12 hours of stimulation, we detected a clear increase in expression from the *CYCB1;1* promoter in Sortin2-treated seedlings but not in control seedlings (**Figure 1B**, 12 h). Importantly, we detected activation of the mitotic reporter exclusively at the pericycle cell layer, suggesting a particular effect on those cells along the primary root that promotes LRFC specification (**Figure 1B**). Indeed, this result rules out a general or nonspecific effect of Sortin2 on cell division and/or mitosis. After 24 hours of treatment, the induction of the p*CYCB1*::GUS reporter was stronger in Sortin2-stimulated seedlings than in control seedlings, and GUS staining was detectable on both sides of the root next to the vascular tissue (**Figure 1B** and **Supplementary Figure 1**), concordantly with the pattern of emerged LR developed by Sortin2 (Pérez-Henríquez et al., 2012). The GUS signal was still restricted to pericycle cells, supporting the specificity of Sortin2 effect. Stimulating seedlings with Sortin2 for 24 and 48 hours increased the number of p*CYCB1*-positive events at the inner cell layer of the main root by 40% and 58%, respectively, compared to control seedlings (**Figure 1C**).

Overall, Sortin2 induces protein membrane trafficking towards the vacuole and induction of cell division events in the pericycle cell layer, strongly suggesting that the Sortin2-induced key event in LR formation targets an early step in LRI, leading to root branching.

### Endocytic trafficking induction induces pericycle cell differentiation to LRFC

**Figure 2:**
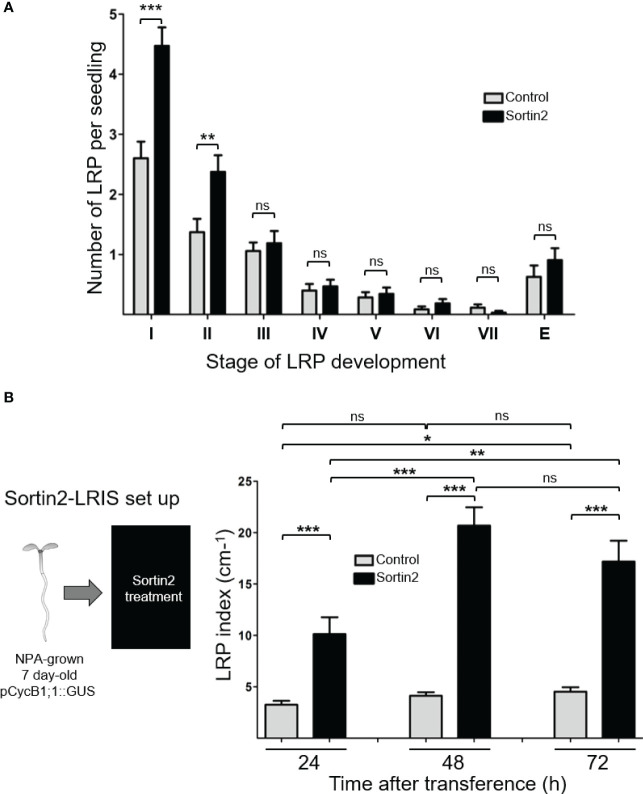
Sortin2 promotes lateral root primordium (LRP) initiation. **(A)** Seven-day-old wild-type (Col-0) seedlings were treated with 25 µg/mL Sortin2 (black bar) for 24 hours or untreated (control; gray bars). The LRPs were scored and classified into stages from I to VII according to Malamy and Benfey (1997). Emerged LR were also scored (E). Results are from three independent experiments (*n* ≥ 32 seedlings). **(B)** A lateral root-inducible system (LRIS) was used to obtain seedlings without pre-formed LRP. p*CYCB1;1*::GUS seeds were sown in NPA for 7 days. Afterwards, NPA-grown p*CYCB1;1*::GUS seedlings were treated with Sortin2 (black bar) for 24, 48, and 72 hours or untreated (controls; gray bars), and the LRP index was evaluated. The results from five experimental replicates (*n* ≥ 26 seedlings) are shown. **(A, B)** The mean and standard error are shown. The results were analyzed using Student’s *t*-tests; brackets denote statistical differences between conditions (*p < 0.05; **p < 0.01; ***p < 0.001; ns, not significant).

To further examine whether that Sortin2 affects an early step of LRI, we evaluated the primordium stages after chemical stimulation (**Figure 2A**). Seven-day-old Arabidopsis seedlings displayed emerged roots and LRPs at all different stages of development (Malamy and Benfey, 1997). After 24 hours, Sortin2-treated seedlings had significantly more stage I and stage II LRPs than control seedlings along the root (**Figure 2A**). However, there was no difference in the number of emerged LRs, ruling out an effect of Sortin2 on the development of previously formed LRPs (**Figure 2A**). This result confirmed that Sortin2 induces the formation of new LRP events by stimulating an early step of LRP organogenesis.

The specificity of Sortin2 on endomembrane trafficking has been thoroughly documented (Zouhar et al., 2004; Norambuena et al., 2008; Pérez-Henríquez et al., 2012; Vásquez-Soto et al., 2015). We previously reported that Sortin2 induces LR formation through a mechanism that depends on its effect on membrane trafficking (Pérez-Henríquez et al., 2012). To test whether the induction of LRPs by Sortin2 depends on endocytic trafficking toward the vacuole, we evaluated the effect of the trafficking inhibitor wortmannin. Indeed, the LRP formation was abolished by wortmannin (**Supplementary Figure 2**). Therefore, we conclude that the induction of endomembrane trafficking toward the vacuole stimulates the mechanism responsible for the formation of new LRPs. Wortmannin exclusively affected Sortin2-triggered LRPs without affecting endogenous LR formation (**Supplementary Figure 2**). Therefore, Sortin2 acts as an agonist of a pathway that is different from the endogenous LRP formation pathway in Arabidopsis.

We used the LRIS (Himanen et al., 2002) to evaluate whether Sortin2 stimulation generates new LRP events on seedlings lacking pre-initiated LRPs (**Figure 2B**). In the LRIS, seedlings are generated in the presence of the auxin transport inhibitor NPA, which inhibits asymmetrical LRFC division, consequently abolishing the endogenous pre-patterning mechanism (Casimiro et al., 2001; Vanneste et al., 2005; Van Norman et al., 2013). In NPA-grown *pCYCB1;1*::GUS seedlings, Sortin2 induced twice as many LRP than developed under the control conditions within the first 24 hours of treatment (**Figure 2B**). These results confirm that endocytic trafficking induction positively targets an event before LRI. Notably, treatment for 48 hours further increased the LRP index (by about 400%, **Figure 2B**) without causing an increase in emerged LRs compared to that in control LRIS seedlings (**Supplementary Figure 3**). Taking into account the timing of LR emergence (about 42 h) (Peret et al., 2012), this result indicated that Sortin2 was unable to accelerate emergence of LRP initiated immediately after the NPA inhibition was released (**Supplementary Figure 3**). However, 72 hours of stimulation led to a 4.6-fold increase in the density of emerged LRs in Sortin2-treated seedlings compared with in control seedlings (**Supplementary Figure 3**). This strongly supports successful LR organogenesis of the Sortin2-induced LRPs in the Sortin2-LRIS. Therefore, Sortin2 RSA remodeling was due to the generation of new LRP events and not a consequence of accelerating development of early-stage LRPs.

**Figure 3:**
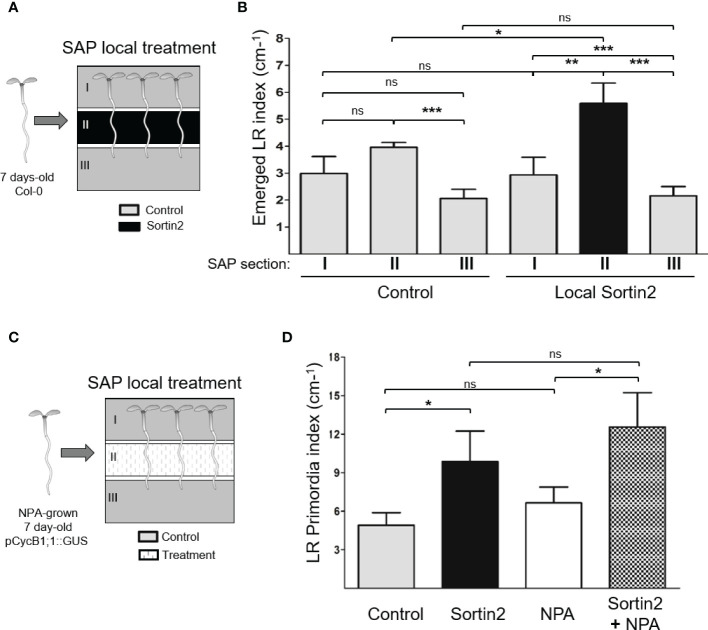
Induction of endomembrane trafficking toward the vacuole induces differentiation of pericycle cells to lateral root founder cells. **(A)** Segmented agar plates (SAPs) were used for local treatments, as illustrated schematically. Section II contained growth medium with Sortin2 (local Sortin2) or DMSO (1%; control), while sections I and III contained growth medium with DMSO. Sections were separated by 2 mm of air. Seven-day-old wild-type (Col-0) seedlings were transferred to SAPs. **(B)** After 6 days, the emerged lateral root of these seedlings were quantified on each SAP section. The assay was performed in triplicate (*n* ≥ 29 seedlings). **(C)** SAPs were established with growth medium supplemented with Sortin2, NPA (10 µM), Sortin2 plus NPA, or DMSO (1%) in section II; sections I and III contained medium with DMSO. Then, seven-day-old NPA-grown p*CYCB1;1*::GUS seedlings were transferred to the plates. **(D)** After 6 days, the GUS-positive lateral root primordia that developed in section II were quantified. Results from three experiments (*n* = 14 [Control], 13 [Sortin2], 8 [NPA], 11 [NPA+Sortin2] seedlings) are shown. **(B, D)** Results were analyzed by Student’s *t*-tests; brackets denote statistical differences between conditions (*p < 0.05; **p < 0.01; ***p < 0.001; ns, not significant).

Different steps in LR organogenesis occur along particular developmental root zones. LR priming takes place in the primary root basal meristem as the previously generated primordium crosses the cell layers at the root differentiation zone and finally emerges (Malamy and Benfey, 1997; Moreno-Risueno et al., 2010; Van Norman et al., 2013; Möller et al., 2016). To investigate whether Sortin2 stimulation could differentiate pericycle cells to LRFC, we applied local treatments at the primary root, far from the basal meristem (**Figure 3**). We used SAPs for local application of Sortin2 to a particular section of the differentiation zone of the primary root (section II), while the rest of the seedling, located in sections I and III, was exposed to control conditions (**Figure 3A** and **Supplementary Figure 4**). Local stimulation of 7-day-old seedlings with Sortin2 induced the formation of LRs, particularly in the section where the chemical was applied (section II, **Figure 3B** and **Supplementary Figures 4, 5**). Therefore, Sortin2 most likely acts directly on the exposed cells rather than triggering a long-distance signal. Indeed, local treatment with Sortin2 in SAPs did not stimulate LR formation in regions exposed to control conditions (sections I and III, **Figure 3B**). The apical root meristem in section III was unaffected, even though this is where LRFC specification and LRI take place. The Sortin2 stimulation of LR formation far from the basal meristem strongly suggests that Sortin2 affects pericycle cell differentiation (**Figure 3B**), inducing *de novo* LR organogenesis.

To validate *de novo* LRP induction by Sortin2, we stimulated NPA-grown p*CYCB1;1*::GUS seedlings in the primary root differentiation zone (section II of the SAPs) with Sortin2 (Sortin2-LRIS; **Figure 3C**). We evaluated the formation of LRP by scoring GUS-positive events in the primary root exposed to each SAP section (**Supplementary Figure 5**). Sortin2 applied in the root differentiation zone of NPA-grown seedlings induced a higher number of new LRP events by 6 days after the release of NPA inhibition than that in NPA-grown seedlings without Sortin2 treatment (**Figure 3D**). The *de novo* developed LRPs were induced in the same section where Sortin2 was added, far from the tissue where LR priming occurs (Van Norman et al., 2013). Sortin2 treatment also induced more emerged LRs compared to control conditions (**Supplementary Figure 5B**), consistent with the results of **Figure 2B**. The application of NPA in section II diminished the index of emerged LRs in sections I and II of seedlings as described by Reed et al. (1998), indicating the effectiveness of the treatment (**Supplementary Figure 5B**). However, NPA did not inhibit the effect of Sortin2 in LRP formation when both were applied locally on section II (**Figure 3D**). Therefore, the mode of action of Sortin2 in LRFC differentiation does not require functional auxin polar transport, which is consistent with the evidence that NPA does not inhibit Sortin2-induced LR formation (Pérez-Henríquez et al., 2012).

Overall, our results indicate that Sortin2-induced endocytic trafficking triggers *de novo* formation of LRPs by stimulating pericycle cell differentiation by a molecular event in which functional polar auxin transport is dispensable.

### Endocytic trafficking targets a mechanism upstream of auxin modules AUX/IAA14 and AUX/IAA28, requiring the SCF complex to induce LRFC specification

**Figure 4:**
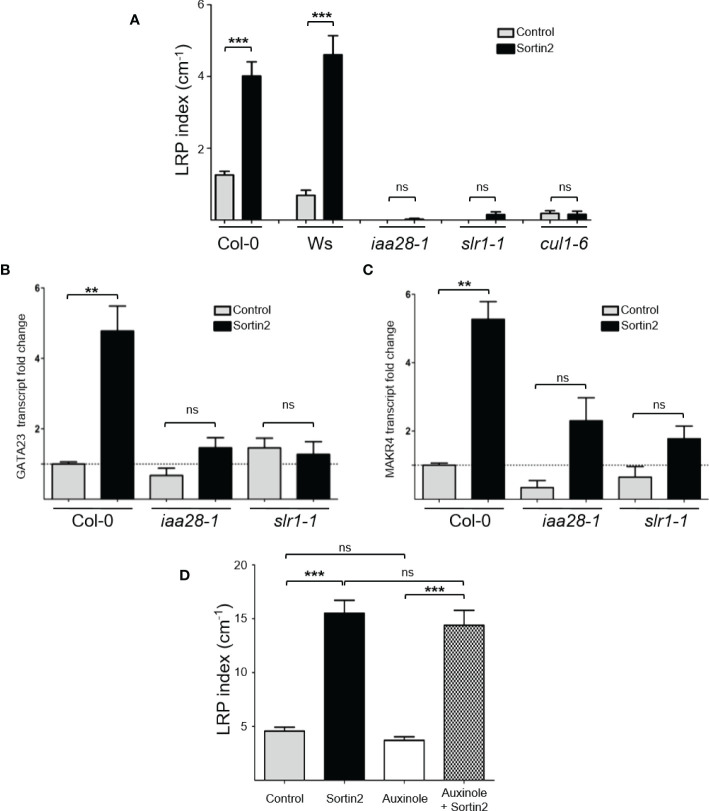
Endomembrane trafficking induction of lateral root founder cell specification occurs upstream of AUX/IAA28 and AUX/IAA14 degradation and depends on CUL1, a SCF complex component. **(A)** Seven-day-old seedlings of the wild types (Col-0 and Ws) and the mutants *iaa28-1*, *slr1-1*, and *cul1-6* were transferred to control (gray bar) or Sortin2 (black bar) treatment conditions. The density of lateral root primordia (LRPs) was evaluated after 72 hours in three independent experiments (*n* ≥ 26 seedlings). **(B, C)** The transcript levels of *GATA23* **(B)** and *MAKR4* **(C)** were evaluated in 7-day-old seedlings of Col-0, *iaa28-1*, and *slr1-1* after 24 hours of Sortin2 treatment. RNA was isolated from a pool of seedlings (*n* ≥ 60) from three independent experiments, and transcript levels of lateral root related genes and reference genes were evaluated by RT-qPCR. Shown are transcript levels of biological triplicates relative to the level in Col-0 seedlings in control conditions (dashed line). The results were analyzed using Student’s *t*-tests and Wilcoxon tests (**p < 0.01; ***p < 0.001; ns: not significant). **(D)** The effect of auxinole (20 μM) on Sortin2-induced LRP formation. Seven-day-old p*CYCB1;1*::GUS seedlings were treated with Sortin2, auxinole, and Sortin2 plus auxinole, and the number of LRPs was scored after 5 days. Results from three experimental replicates are shown (*n* = 25 [Control], 35 [Sortin2], 15 [NAA], 35 [auxinole], 12 [NAA+auxinole], 32 [Sortin2+auxinole] seedlings). Results were analyzed using Student’s *t*-tests; brackets denote statistical differences between conditions (**p < 0.01; *** *p* < 0.001; ns, not significant).

The SCF^TIR1/AFBs^ complex mediates the degradation of the AUX/IAA transcriptional inhibitors leading to auxin-dependent transcription and, subsequently, different physiological and developmental processes (Mockaitis and Estelle, 2008; Salehin et al., 2015; Weijers and Wagner, 2016). Auxin-signaling modules led by AUX/IAA28 and AUX/IAA14 drive LR formation mediated by endogenous auxin regulating LRFC priming and LRI, respectively (Rogg et al., 2001; Fukaki et al., 2002). The gain-of-function mutants *iaa28-1* and *slr1-1* are unable to develop LRPs since the AUX/IAA28 and AUX/IAA14 proteins cannot be degraded (Rogg et al., 2001; Fukaki et al., 2002). To test the requirement of these two auxin modules in Sortin2-mediated LRP formation, we challenged both mutants with Sortin2. Indeed, Sortin2 was unable to induce the formation of LRPs in these two mutants (**Figure 4A**), suggesting that the endocytic inducer Sortin2 targets a molecular component upstream of both AUX/IAA28 and AUX/IAA14.

So far, the earliest event described for LR priming is the transcriptional activation of *GATA23* driven by AUX/IAA28 auxin-mediated signaling (De Rybel et al., 2010). Downstream of *GATA23*, *MAKR4* is also transcriptionally activated in the LRFC, resulting in LRI (De Rybel et al., 2010; Xuan et al., 2015). Treatment with Sortin2 resulted in induction of both *GATA23* and *MAKR4* after 24 hours (**Figures 4B, C**), which is consistent with the induction of LRFC specification (**Figure 3**). However, the treatment did not induce the transcript levels of either *GATA23* or *MAKR4* in the mutants *slr1-1* and *iaa28-1* (**Figures 4B, C**). Therefore, Sortin2 targets a pathway upstream of AUX/IAA14 and AUX/IAA28 to induce LR priming.

The SCF complex participates in different plant processes involving hormone signaling, developmental processes, and responses to environmental cues (Hua and Vierstra, 2011). This complex functions as an E3 ubiquitin ligase, with the substrate to be ubiquitinated depending on its F-box protein member. In the case of auxin-mediated signaling, the F-box members are the TIR1/AFB proteins. The auxin receptors TIR1, AFB1, AFB2, and AFB3 are dispensable for Sortin2 bioactivity upon LR formation (Pérez-Henríquez et al., 2012) as well for LRP formation (**Supplementary Figure 6**). Indeed, a specific inhibitor of the function of TIR1/AFBs, auxinole (Hayashi et al., 2008; Hayashi et al., 2012), did not inhibit the development of LRP induced by Sortin2 (**Figure 4D**). The same concentration of auxinole did inhibit LR formation and DR5-auxin responsiveness induced by 1-naphthaleneacetic acid (NAA; **Supplementary Figure 7**), however, as described previously (Hayashi et al., 2012). Therefore, Sortin2 does not require the functionality of any of the TIR1/AFB receptors for inducing LRP formation.

Arabidopsis contains a diverse set of SCF complexes that recognize diverse substrates based on the associated F-box proteins, explaining their specificity (Hua and Vierstra, 2011). An essential component of the SCF complex is CULLIN, a scaffold protein for binding the other two SCF members: ASK1 and RBX1. In Arabidopsis, among the CULLIN proteins, CULLIN1 (CUL1) participates in several SCF complexes (Hua and Vierstra, 2011). The *CUL1* partial loss-of-function mutant *cul1-6* results in defects of LRI, indicating that *CUL1* has a role in LR formation (Moon et al., 2006). To test whether the SCF complex is required for LRFC differentiation induced by the endocytic trafficking pathway, we challenged *cul1-6* with Sortin2. However, Sortin2 did not induce LRP in *cul1-6* after 72 hours of treatment (**Figure 4A**). Thus, endocytic trafficking–dependent LRFC differentiation requires a functional SCF.

As mentioned before, the TIR1/AFB family of auxin receptors was not required for Sortin2-induced LRP formation (**Figure 4**). However, the degradation of AUX/IAA28 and AUX/IAA14 is required for the Sortin2 mechanism to transcriptionally activate downstream GATA23 and MARK4. Therefore, the AUX/IAA28 and AUX/IAA14 degradation required for Sortin2 bioactivity in LRFC differentiation most likely is executed by an alternative F-box protein of the SCF complex or else by an unknown mechanism. In Arabidopsis, about 700 F-box proteins have been identified (Gagne et al., 2002), making it difficult to predict the F-box(s) participating in Sortin2-induced LRFC differentiation. We selected several F-box proteins in which the corresponding loss-of-function mutant displays an altered LR formation phenotype and challenged them with Sortin2 to evaluate LRP formation. Among the tested mutants, the loss of function of *CEG* (Dong et al., 2006), *SKP2a* (Jurado et al., 2008), and *SKP2b* (Manzano et al., 2012) resulted in resistance to Sortin2, suggesting that their protein products might be involved in the effect of Sortin2 on LR branching (**Supplementary Figure 8**); they are likely required for the endocytic trafficking–based LRP formation mechanism. However, further investigation is needed to elucidate their function more precisely. In addition, the participation of other F-box(s) in SCF signaling should be investigated.

Overall, we conclude that the mechanism of lateral root founder cell specification mediated by endocytic trafficking is upstream of AUX/IAA28 and GATA23 and requires a functional SCF complex.

### Endocytic trafficking induction defines a distinctive mechanism of LRFC specification that requires Ca^2+^ uptake

**Figure 5:**
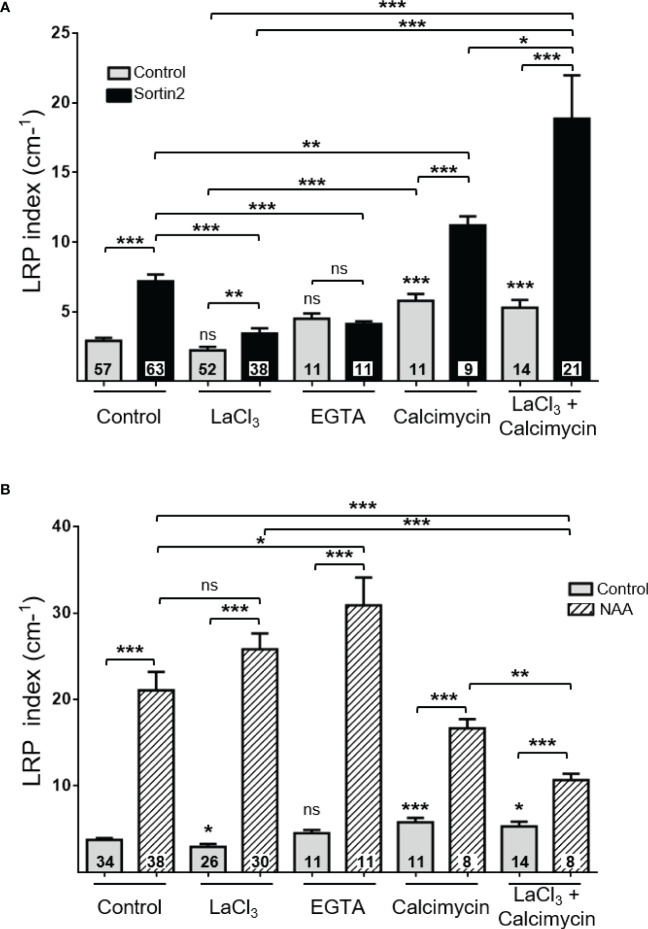
The requirement for extracellular calcium uptake reveals a distinctive lateral root initiation mechanism induced by endocytic trafficking from auxin NAA induction. Induction of lateral root primordia by Sortin2 **(A)** and NAA (1 μM; **B**) in the presence of LaCl_3_ (1 mM), EGTA (10 mM), the ionophore calcimycin (80 µM) or calcimycin plus LaCl_3_ (1 mM) for 72 hours in 7-day-old Col-0 seedlings. Numbers inside bars indicate the number of seedlings scored in three independent experiments. One-way ANOVA and Tukey’s *post-hoc* test were performed. Statistically significant differences between the control condition and a specific treatment are indicated above each bar. Brackets denote statistical differences between conditions (*p < 0.05; **p < 0.01; ***p < 0.001; ns, not significant).

**Figure 6:**
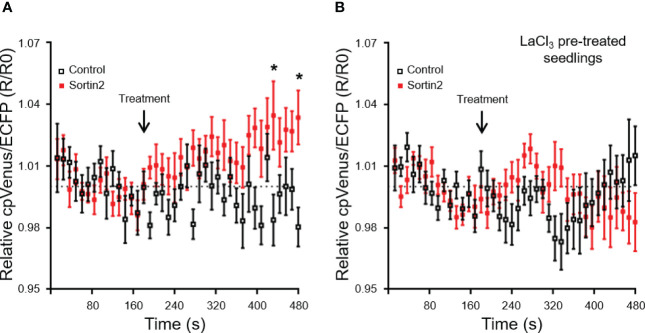
Sortin2 treatment increases cytoplasmic Ca^2+^ levels in the primary root. **(A)** Five-day-old seedlings of the cytoplasmic Ca^2+^ reporter line NES-YC3.6 were incubated in a perfusion chamber. ECFP and cpVenus fluorescence in the root differentiation zone was analyzed by confocal microscopy to determine the cytoplasmic Ca^2+^ level. Seedlings were incubated with MSL containing 1% DMSO (time 0) and imaged for 180 seconds. Then, seedlings were treated with MSL supplemented with Sortin2 (red) or 1% DMSO (control, black) and recorded for 300 seconds. **(B)** Five-day-old seedlings were treated with LaCl_3_ (1 mM) for 1 hour before being placed in the chamber. The treatment and recording were performed in conditions similar to those described in **(A)** The normalized ECFP/cpVenus ratio from three independent experiments is shown (*n* = 12 seedlings). The dashed line indicates a normalized ratio value of 1.0. Results were analyzed using two-way ANOVA with Sidak *post-hoc* tests. Only statistically significant differences between the Sortin2 and control treatments are indicated (*p < 0.01).

Calcium has been proposed as a signal for LR formation driven by stimuli where TIR/AFB auxin receptors are dispensable. Mechanical root stimulation, which promotes LR formation, rapidly increases cytoplasmic Ca^2+^ levels in different primary root cell layers, including the pericycle and endodermis (Richter et al., 2009). Blocking extracellular Ca^2+^ influx with LaCl_3_ results in the inhibition of LR formation induced by root bending (Richter et al., 2009). To test whether Ca^2+^ is involved in Sortin2-induced LRP formation, we evaluated the effect of LaCl_3_ on this process. LaCl_3_ significantly reduced Sortin2 LRP induction when seedlings were co-treated with both Sortin2 and LaCl_3_ for 72 hours (**Figure 5A**). Pretreatment with LaCl_3_ for 1 hour was enough to suppress the response to Sortin2 in terms of LRP formation (**Supplementary Figure 9**). The calcium blockers verapamil and nifedipine, unlike LaCl_3_, did not interfere with Sortin2 induction of LRP formation (**Supplementary Figure 10**). Chelating extracellular Ca^2+^ using EGTA had a similar effect to that of LaCl_3_, strongly suggesting that calcium uptake from the cell wall or apoplast is involved in the cellular mechanism induced by Sortin2 for LR formation (**Figure 5A**). Consistent with this hypothesis, the Ca^2+^ ionophore calcimycin, which increases cytoplasmic Ca^2+^ (Monshausen et al., 2008), completely abolished the LaCl_3_ inhibition over Sortin2 LRP induction (**Figure 5A** and **Supplementary Figure 9**). This result supports the idea that an increment of cytoplasmic Ca^2+^ is required for LRP formation triggered by Sortin2. Indeed, Sortin2 increased the cytoplasmic Ca^2+^ level in the differentiation zone of the primary root (**Figure 6A**), and this rise in Ca^2+^ was impaired by LaCl_3_ (**Figure 6B**). Moreover, pretreatment of seedlings with LaCl_3_ was enough to suppress the response to Sortin2 in terms of LRP formation (**Supplementary Figure 9**). Therefore, a cytoplasmic Ca^2+^ increase is essential for LRP formation triggered by the induction of endocytic trafficking. This increase is due to Ca^2+^ uptake from the extracellular space.

In contrast, NAA-induced LRP formation did not require Ca^2+^ uptake, as neither LaCl_3_ nor EGTA inhibited the formation of LRPs stimulated exogenously by NAA (**Figure 5B**). On the other hand, altering cytosolic Ca^2+^ impacted both Sortin2- and NAA-induced LR emergence (**Supplementary Figure 11**). The different requirements on calcium flux show that stimulating endocytic trafficking with Sortin2 induces a distinctive mechanism from the canonical auxin-mediated LRP formation.

## Discussion

**Figure 7:**
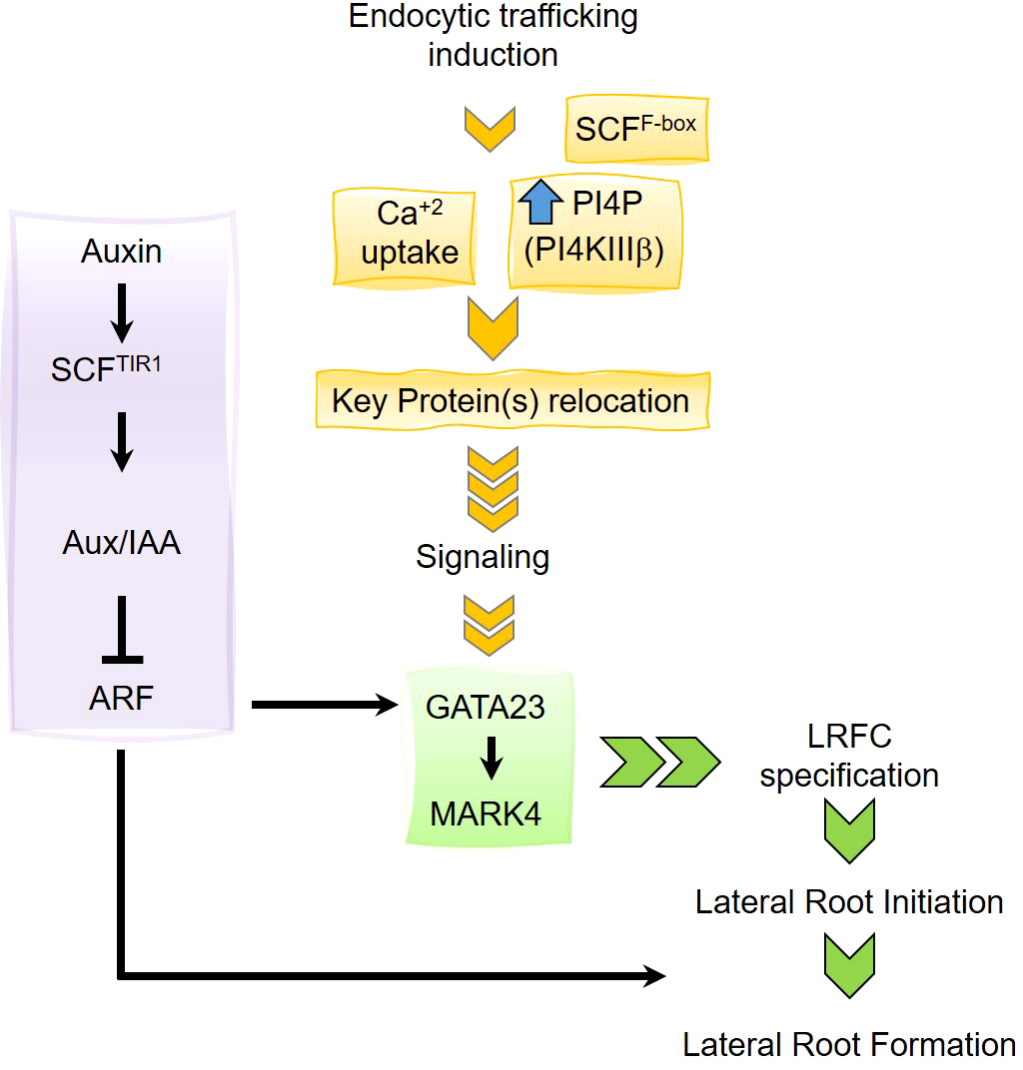
Working model. The lateral root organogenesis driven by endocytic trafficking to the vacuole requires the increase of phosphatidylinositol 4-phosphate (PI4P) level mediated by the phosphatidylinositol 4-kinases PI4KIIIβ1 and PI4KIIIβ1 (Rubilar-Hernandez et al., 2019). Relocation of key protein within the endomembrane system would induce a signaling cascade promoting the induction of GATA23 and consequently activation of MARK4 to promote lateral root founder cell (LRFC) specification. The LRFC specification driven by the induction of endocytic trafficking requires Ca^+2^ entrance and the function of a SCF complex although the F-Box is unknown yet. This pathway is distinctive from the auxin-driven lateral root primordia development.

LRFCs undergo a developmental program to form LR. It is known that certain pericycle cells are specified to become LRFCs; however, the specification process has been poorly understood. In this article, we report that stimulation of endocytic trafficking toward the vacuole using the chemical Sortin2 positively impacts LRFC specification (**Figure 7**). Chemical induction of the endocytic pathway triggers *de novo* and local LRP formation, leading to functional LR organogenesis. Consistent with this, the developmental stimulus of Sortin2 is located upstream of molecular events defining LRFC activation, such as *GATA23* and *MAKR4* induction. Moreover, the endocytic trafficking–induced mechanism requires calcium influx and a functional SCF complex, suggesting a signaling process involved in the induction of organogenesis. This mechanism is distinctive from endogenous LR formation. This report provides the first evidence of a cellular process preceding the specification of the LRFCs.

### The endocytic pathway induces activation of LRFCs by a distinctive mechanism other than endogenous LR formation

Our current results demonstrate precisely that Sortin2-induced LR formation is due to an increase in the formation of new primordia due to the induction of LRFC specification (**Figures 1**, **3**, **5**). Genetic and chemical inhibition of TIR1/AFBs revealed their dispensability in the action of Sortin2 in promoting LRP formation (**Figure 4**). These results suggest a molecular mechanism distinct from the well-described auxin-mediated LR formation (Reed et al., 1998; Dharmasiri et al., 2005; Xuan et al., 2015). Furthermore, differences in calcium requirements substantially distinguish these two mechanisms influencing LRP formation (**Figure 5**). Both chemical inhibition of TIR1/AFBs and perturbation of calcium homeostasis affected the Sortin2 mechanism in a different manner than the application of NAA and endogenous LRP formation (**Figures 4**, **5**). Endogenous LRP formation was only slightly inhibited by LaCl_3_; however, this effect was unlikely to be related to calcium uptake since the calcium chelator EGTA did not produce the same effect as LaCl_3_ (**Figure 5**). Endogenous LRP formation requires a functional polar auxin transport along the primary root (Reed et al., 1998; Casimiro et al., 2001). In contrast, local stimulation with Sortin2 successfully drove LRFC priming even in the absence of auxin polar transport (**Figure 3**). Importantly, Sortin2 does not act as an auxin signaling agonist (Pérez-Henríquez et al., 2012). Therefore, Sortin2 stimulates LRFC specification in a fashion, without inducing canonical auxin signaling or the redistribution or accumulation of auxin along the primary root.

Nevertheless, the mechanism driven by endocytic trafficking toward the vacuole that induces LRP formation requires auxin signaling later on. AUX/IAA14 is required for Sortin2-induced LRP formation (**Figure 4**). *slr-1* mutants are resistant to Sortin2 stimulation (**Figure 4**) as well as to NAA (Fukaki et al., 2002), suggesting that the pathways induced by Sortin2 and auxin are connected (**Figure 7**). Although Sortin2 stimulation triggers activation of *GATA23* and *MARK4*, consistent with the induction of LRFC specification, this mechanism requires AUX/IAA28 degradation (**Figure 4**), suggesting that stimulating endocytic trafficking initiates an organogenesis program carried out by molecular players, such as AUX/IAA28, that also respond to auxin (De Rybel et al., 2010). The activation of *GATA23* mediated by AUX/IAA28 is driven by auxin as part of the endogenous program of LR organogenesis (De Rybel et al., 2010). Our data suggest that the molecular components upstream of AUX/IAA28, which have yet to be described, are different from the determinants of the mechanism mediated by endocytic trafficking.

### Endocytic trafficking to the vacuole triggers LRFC specification

Bioactive chemicals affecting endomembrane trafficking result in developmental and physiological responses, allowing pathway dissection as well as identification of molecular players (Norambuena and Tejos, 2017). Sortin2 has a precise cellular effect on endomembrane trafficking (Zouhar et al., 2004; Pérez-Henríquez et al., 2012; Vásquez-Soto et al., 2015). The fact that wortmannin abolished Sortin2-induced LRP formation indicates that chemical stimulation is a convenient agonist of the mechanism that depends on trafficking toward the vacuole (**Supplementary Figure 2**). Sortin2 induces the endocytic trafficking from the PM to the vacuole in 2 to 6 hours, before cell division (**Figure 1**). p*CYCB1;1*::GUS activation is restricted to pericycle cells, demonstrating the specificity of the Sortin2 mechanism on LR organogenesis. The induction of cell division without pericycle cell fate specification would not result in LRI (Vanneste et al., 2005). Therefore, Sortin2-induced cell division is most likely due to the normal progress of a differentiated LRFC toward the LR developmental program. Sortin2 does not induce general cell division, since reporters were not detected in other primary root cell layers and both the root structure and number of cell layers were normal under Sortin2 treatment (**Figure 1**).

The distinctive mechanism induced by the endocytic pathway could be activated by physiological or environmental stimuli. The role of calcium as a second messenger is broadly recognized in myriad processes in plants. Calcium content in the cytoplasm probably increases rapidly after Sortin2 application (**Figure 6**). The calcium required for Sortin2-induced LR formation is incorporated from the extracellular environment by LaCl_3_-sensitive channels (**Figure 5**). Consistent with this, roots display the same sensitivity to calcium influx blockers in response to mechanic stimulation of LR development (Monshausen et al., 2009; Richter et al., 2009), strongly suggesting that Sortin2 stimulation mimics the effect of this stimulus. Primary roots sense water availability in the rhizosphere, and this dramatically affects RSA (Bao et al., 2014). LRs are predominantly formed in the direction of water-rich patches of soil, confirming that the LR pattern is indeed modified according to environmental conditions. However, the mechanisms whereby the hydraulic conductivity signal is sensed and transduced remain unknown.

The SCF complex works as an E3 ubiquitin ligase, mediating ubiquitination and polyubiquitination of substrates either for degradation in the proteosome, as for AUX/IAA proteins, or for protein internalization and trafficking to the vacuole (Hua and Vierstra, 2011). The target of such a complex depends on the F-box protein that binds to the complex. Although TIR1/AFB F-box proteins are dispensable for the Sortin2 mode of action, a functional SCF complex is required (**Figure 4**). As discussed above, Sortin2 stimulates a mechanism upstream of IAA28 degradation. Therefore, the SCF complex could be required strictly for AUX/IAA degradation or for ubiquitination or polyubiquitination of a different type of substrate (see below).

### Endocytic trafficking could participate in a signaling mechanism for LRFC specification

Arabidopsis probably does not sense Sortin2 as general stress signal since seedlings do not show subcellular or physiological detrimental phenotypes (Pérez-Henríquez et al., 2012). Instead, Sortin2 could mimic a stimulus that turns on a mechanism mediated by a PM- or endosome-associated signaling molecule that is being relocated within the endomembrane system. Such a molecule, still unidentified, would establish a switch in the molecular identity of pericycle cells. The convenience of a fast mechanism such as endocytosis would provide quick responses to environmental cues. Therefore, the Sortin2-targeted mechanism could be beneficial under fluctuating conditions that the root faces during the plant lifespan. However, perturbing trafficking toward the vacuole does not affect endogenous LRP formation (**Supplementary Figure 2**). Therefore, it is likely that Sortin2 induces a different pathway from the one established by the DR5-oscillation alternating pattern (Moreno-Risueno et al., 2010). Indeed, the alternating pattern of LR formation can be modified by external stimuli such as root bending (Kircher and Schopfer, 2016). Evidence suggests that both mechanisms coexist, given the RSA output. Therefore, it is possible that external stimuli result in a fast-signaling endocytic trafficking–mediated mechanism for LR organogenesis. Endocytosis and endosomal signaling are widely conserved mechanisms across all life kingdoms (von Zastrow and Sorkin, 2007; Geldner and Robatzek, 2008; Sigismund et al., 2012). In plants, brassinosteroid signaling requires the endocytic trafficking of the PM receptor BRI1, which is finally targeted to the vacuole (Geldner et al., 2007; Martins et al., 2015). BRI1 endocytosis is constitutive and independent of its hormone ligand; however, it is essential for setting the brassinosteroid-led gene program (Geldner et al., 2007). There is also evidence of ligand-regulated endocytosis signaling influencing developmental and physiological responses. Researchers have identified the existence of different classes of small signaling peptides (Czyzewicz et al., 2013; De Coninck and De Smet, 2016). These peptides act as ligands for receptors that transduce particular signals, interfering with signaling cascades or playing a crucial role in cell-to-cell communication (Murphy et al., 2012; Tintor et al., 2013; Qu et al., 2015; Roberts et al., 2016). Endocytosis has been shown to be essential for small-peptide ligand–receptor complex signaling in response to pathogens (Ortiz-Morea et al., 2016). Recently, Roberts et al. (2016) proposed that the small-peptide C-TERMINALLY ENCODED PEPTIDE 5 (CEP5) may interact with the receptor XYLEM INTERMIXED WITH PHLOEM 1 (XIP1)/CEP RECEPTOR 1 (CEPR1) to regulate LRI (Roberts et al., 2016). XIP1 seems to have a role in promoting LR formation. However, whether XIP1 function could be mediated by the rate of endocytosis or relocation remains unknown.

Ligand–receptor endocytosis is regulated by polyubiquitination, targeting the receptor with or without the ligand to the vacuole or lysosome in the case of animal cells (Sigismund et al., 2012). The associated signaling is turned on and the receptor can be degraded once cells start their differentiation or mitotic program. The PM receptor for a small peptide involved in root meristem development has been identified, and its interaction with the small peptide ligand promotes phosphorylation and polyubiquitination of the receptor, in a process resembling features of receptor down-regulation (Ou et al., 2016). Thus, it is possible that the SCF complex dependence of Sortin2-LRFC specification (**Figure 4**) relies on the ubiquitination of receptor–ligand trafficking from the PM to the endosomes and vacuole. However, to test this hypothesis, the putative small peptide and/or receptor(s) should be identified first. On the other hand, endocytosis protein trafficking has been described as a mechanism for regulating the level of nutrient transporters at the PM according to nutrient availability (Takano et al., 2005; Barberon et al., 2011; Bayle et al., 2011). However, downstream signaling associated with an endocytosis-mediated mechanism has not yet been reported. Indeed, these transporters are internalized under sufficient or excess nutrient contents, conditions that do not induce LR branching.

RSA modification requires an efficient system of sensing needs and a rapid response to cope with and overcome adverse situations. For LR formation in response to mechanical stimulation, the existence of a mechanosensor has been proposed; however, molecular candidates to support this hypothesis are lacking (Ditengou et al., 2008; Monshausen et al., 2009). The calcium dependency of Sortin2-induced LRFC specification suggests a role of endocytosis-mediated signaling as a candidate for such stimulus. Whether this is the case will remain unclear until the molecule that is transported to the intracellular compartments can be identified.

## Data availability statement

The original contributions presented in the study are included in the article/**Supplementary Materials**. Further inquiries can be directed to the corresponding author.

## Author contributions

SM-H, CR-H, PP-H designed and performed experiments, prepared the figures, and wrote the draft. LN conceptualized and designed the research, provided supervision, acquired funding and wrote the final manuscript. All authors contributed to the article and approved the submitted version.
