# Supplementary material for: Endocytic trafficking induces lateral root founder cell specification in Arabidopsis thaliana in a process distinct from the auxin-induced pathway
Source: Front Plant Sci. 2023 Jan 16;13:1060021. doi: 10.3389/fpls.2022.1060021 (PMC9885164; doi:10.3389/fpls.2022.1060021)
Supplement: Supplementary file 1 [file Presentation_1.pdf]

Supplementary Material

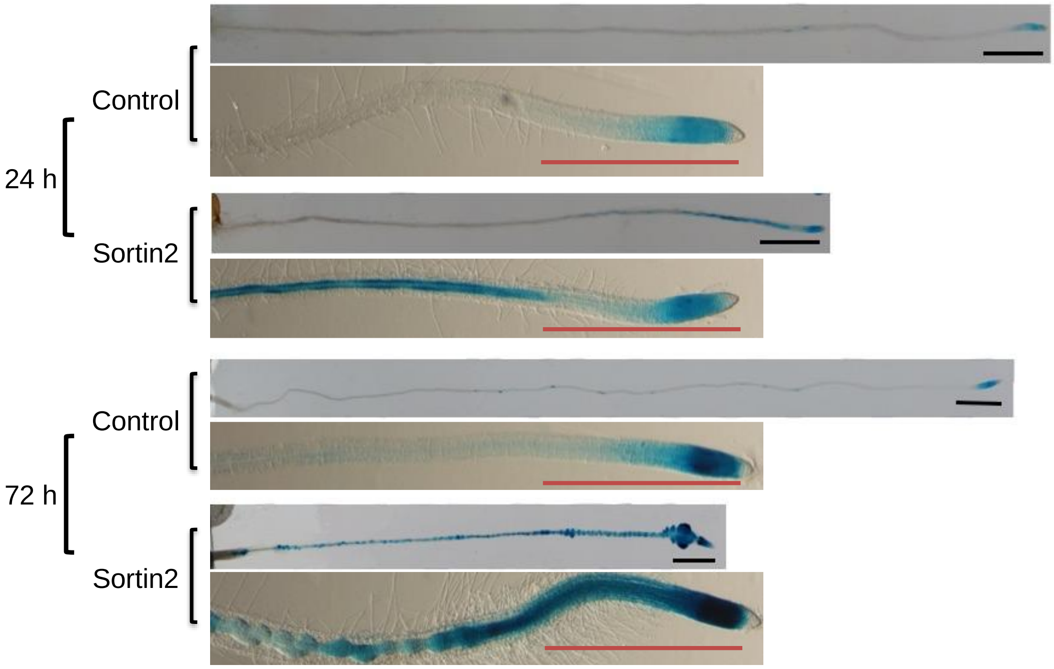

Supplementary Figure 1. Sortin2 promotes positive p*CYCB1;1*::GUS events at pericycle cells Seven-day-old p*CYCB1;1*::GUS reporter line seedlings were treated with Sortin2 for 24 and 72 hours(or not treated, for controls), and their GUS activity was evaluated. Representative brightfield images of GUS activity in seedlings is shown. Scale bar: red = 1mm and black= 2mm.

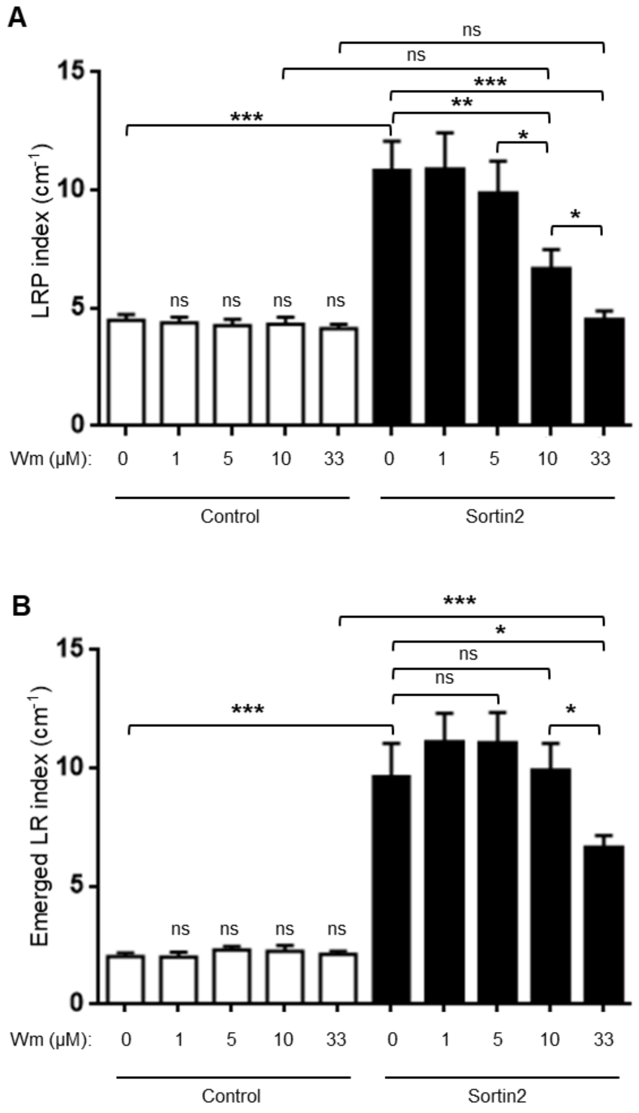

Supplementary Figure 2. The effect of Sortin2 on LRP formation relies on its effect on endocytic trafficking to the vacuole. Seven-day-old Col-0 seedlings were treated with different concentrations of wortmannin (Wm) as indicated, with (black bars) or without (control; white bars) Sortin2, for 72 hours. LRP (A) and emerged LR (B) indexes were quantified. Results from two experimental replicates are shown ($n \geq 11$). One-way ANOVA with Tukey's post-hos tests was used to analyze the results; brackets denote statistical differences between conditions (*$p < 0.05$; **$p < 0.01$; ***$p < 0.001$; ns: not significant).

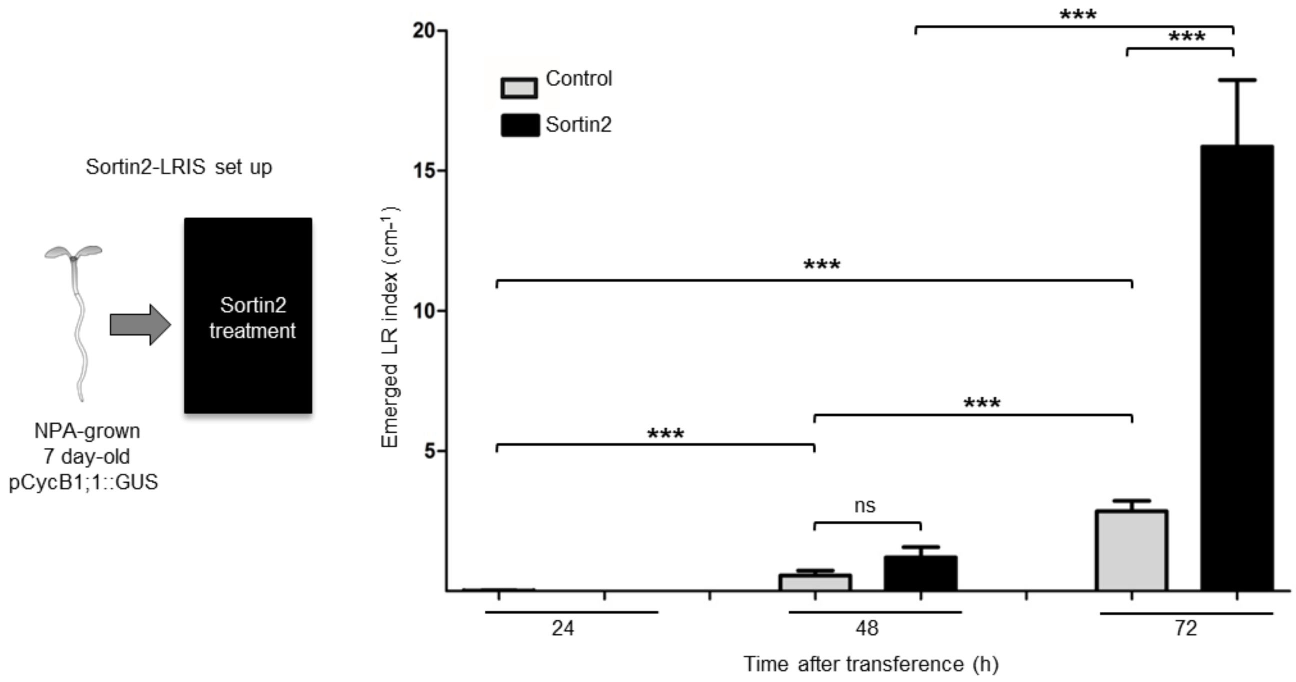

Supplementary Figure 3. The endocytic trafficking inducer Sortin2 promotes organogenesis of lateral roots. Seven-day-old NPA-grown p*CYCB1;1*::GUS seedlings were treated with Sortin2 for 24, 48, and 72 hours. Density of emerged lateral roots under Sortin2 (black bar) and control (gray bar) treatments was evaluated. The results from five experimental replicates ($n \geq 26$ seedlings) were analyzed using Student's *t*-tests; brackets denote statistical differences between conditions (***$p < 0.001$; ns: not significant).

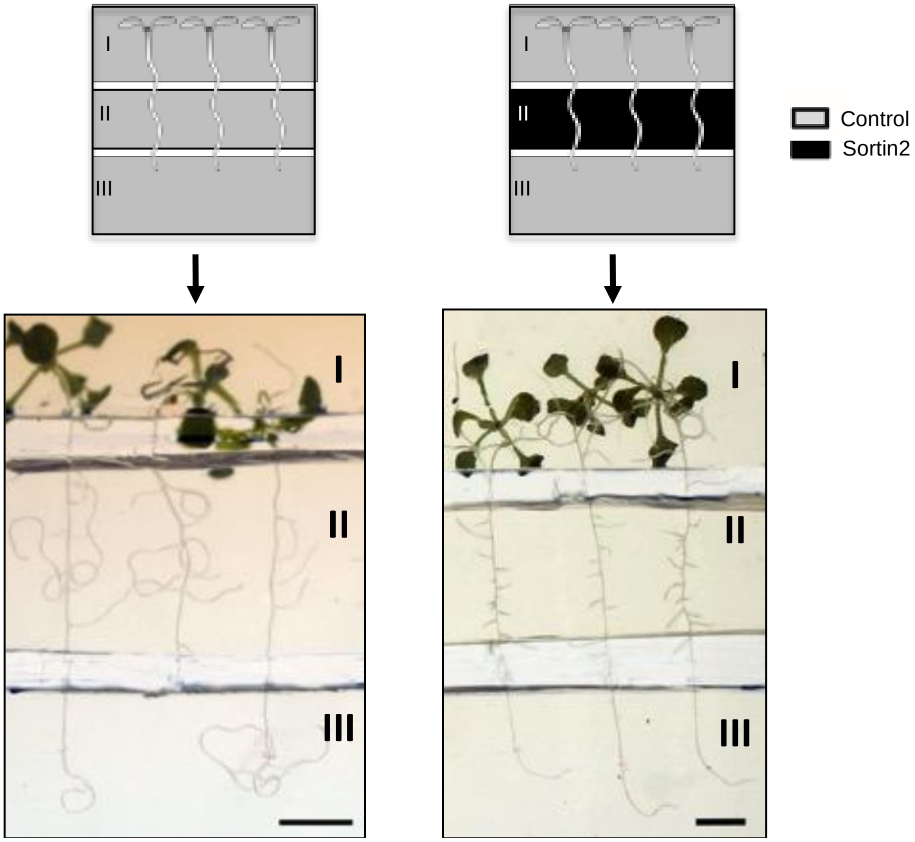

Supplementary Figure 4. Induction of endomembrane trafficking toward the vacuole induces differentiation of pericycle cells to lateral root founder cells. (**A**) Segmented agar plates (SAPs) were used for local treatments, as illustrated schematically. Section II contained growth medium with Sortin2 (local Sortin2) or DMSO (1%; control), while sections I and III contained growth medium with DMSO. Sections were separated by 2 mm of air. Seven-day-old wild-type (Col-0) seedlings were transferred to SAPs. Representative images are shown. Scale bar: 5 mm.

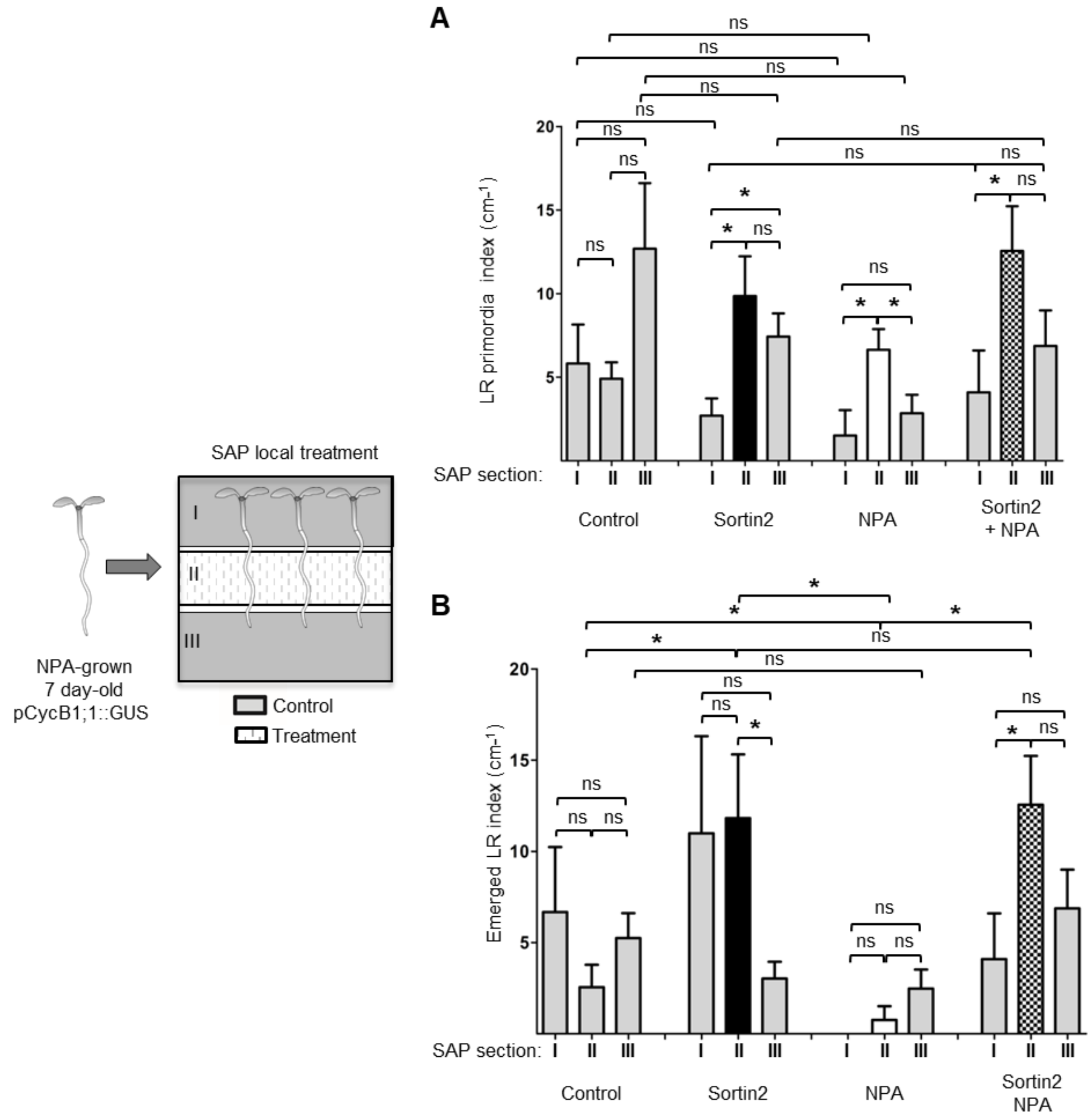

Supplementary Figure 5. Endomembrane trafficking toward the vacuole induces differentiation of pericycle cells to become lateral root founder cells. NPA-grown p*CYCB1;1*::GUS seedlings were transferred to SAPs. GUS-positive lateral root primordia on each section were evaluated after 6 days. The central section (Section II) of the SAP contained DMSO (1%; control), Sortin2, NPA (10 µM), and NPA plus Sortin2, while Sections I and III contained regular medium with DMSO (1%) as the control. Each section was separated from the others by 2 mm of air. LRP (**A**) and emerged LR (**B**) indexes are shown. Results from three experiments ($n \geq 8$ seedlings) were analyzed using Student's *t*-tests; brackets denote statistical differences between conditions (*$p < 0.05$; ns: not significant).

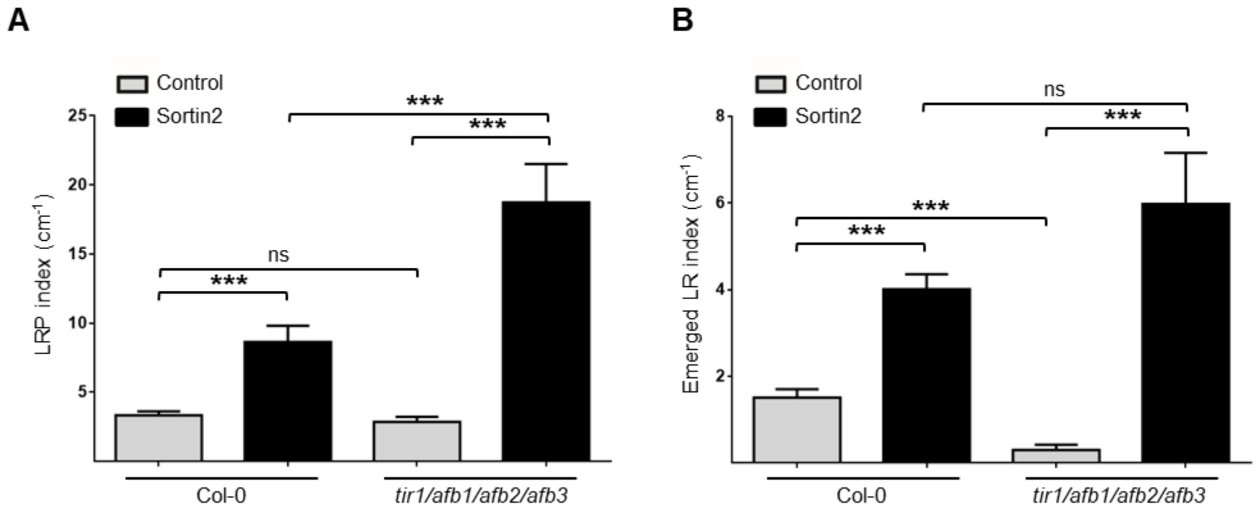

Supplementary Figure 6. TIR1, AFB1, AFB2, and AFB3 are dispensable for LR organogenesis induced by endocytic trafficking. Seven-day-old Col-0 (n = 12) and quadruple loss-of-function mutant *tir1*/*afb1*/*afb2*/*afb3* ($n \geq 9$) seedlings were treated with Sortin2 (black bars) or grown under control conditions (gray bars) for 72 hours. LRP (A) and emerged LR (B) indexes were quantified. Data from two experimental replicates are shown ($n \geq 9$). Results were analyzed using Student's *t*-tests; brackets denote statistical differences between conditions (***$p < 0.001$; ns: $p > 0.05$).

.

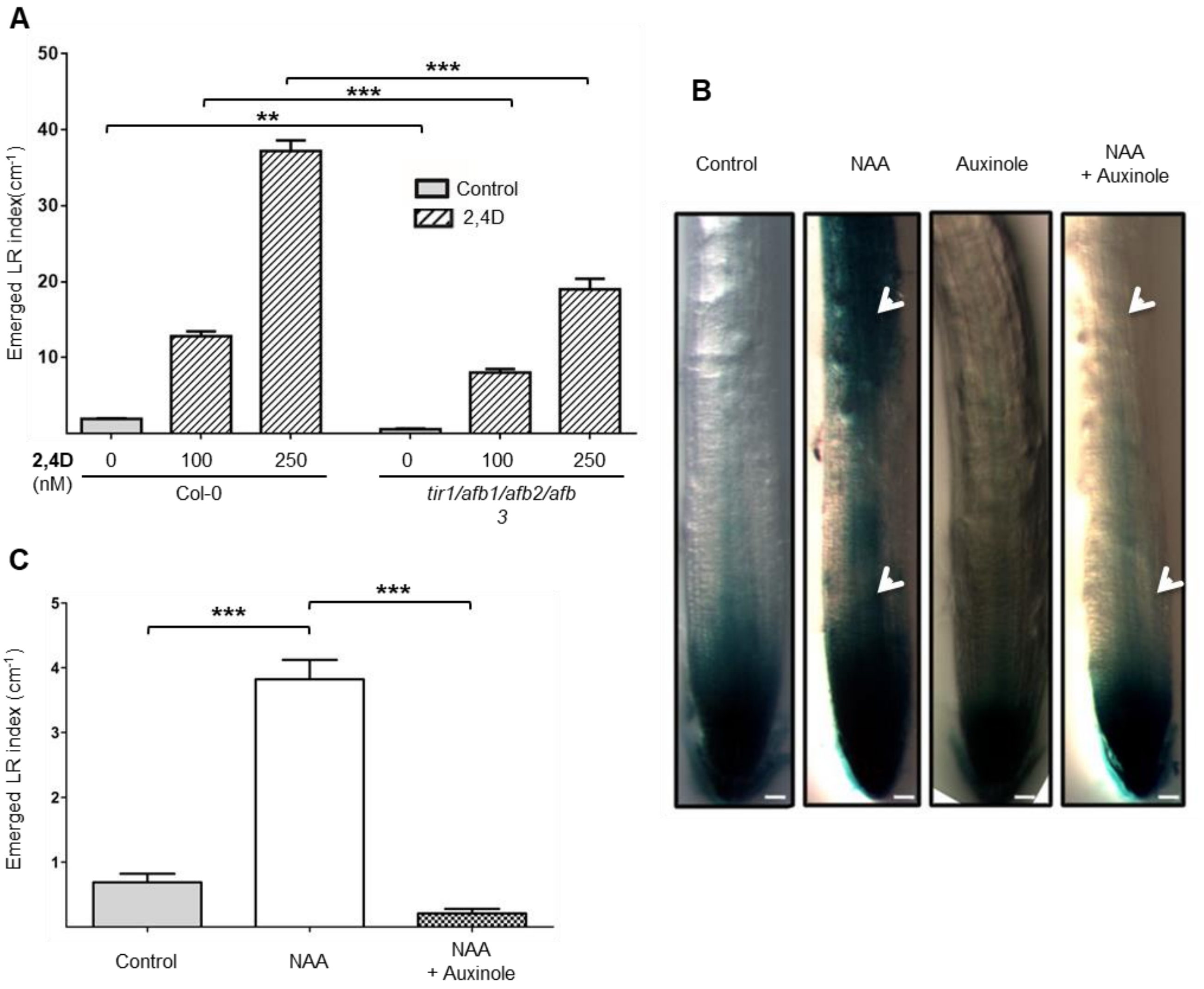

Supplementary Figure 7. Members of the TIR1/AFB auxin receptor family participate in LR formation induced by exogenous auxin-induced. A. Seven-day-old Col-0 and *tir1/afb1/afb2/afb3* seedlings were grown under control conditions (gray bars, no auxin) or with the synthetic auxin 2,4-D (100 and 250 nM, hatched bars) for 5 days. Emerged LRs were then evaluated and are shown as an index from two experimental replicates ($n \geq 18$ Col-0 seedlings; $n \geq 20$ *tir1/afb1/afb2/afb3* seedlings). Results were analyzed with Student's *t*-tests (*$p < 0.05$; **$p < 0.01$; ***$p < 0.001$; ns, not significant). **B**. Seven-day-old seedlings of the auxin-responsive *pDR5*::GUS reporter line were grown under control conditions or were treated with NAA (2 µm), auxinole (20 µM), or both NAA (2 µm) and auxinole (20 µM) for 5 hours. Scale bar, 50 µm. **C**. Seven-day-old seedlings of the p*CYCB1;1*::GUS reporter line were grown under control conditions ($n \geq 8$) or were treated with NAA (0.2 µm; $n \geq 8$) or both NAA (0.2 µm) and auxinole (20 µM; $n = 19$ seedlings) for 5 days. Emerged LRs were evaluated and are shown as an index ($n \geq 19$ seedlings). The results were analyzed using Student's *t*-tests; brackets denote statistical differences between conditions (***$p < 0.001$).

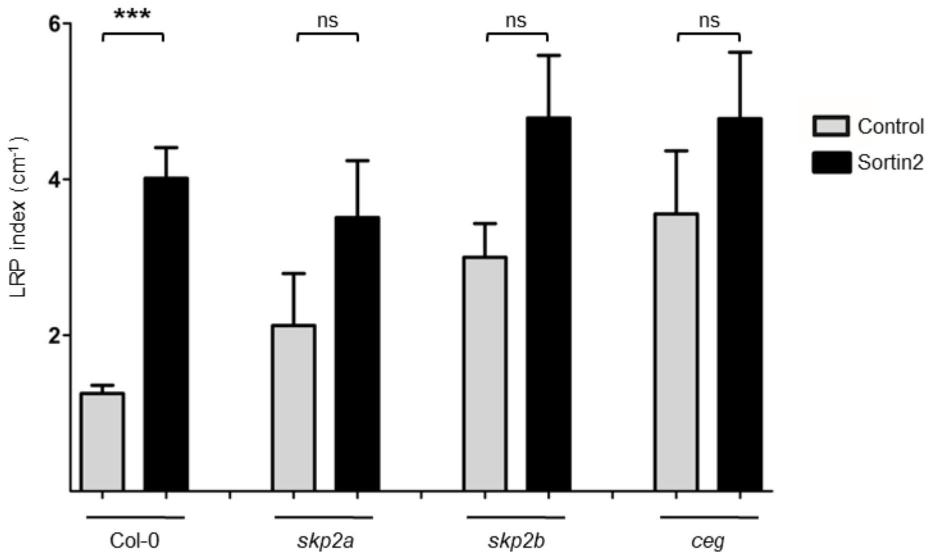

Supplementary Figure 8. F-box proteins are required for Sortin2 induction of LRPs. Seven-day-old wild-type (Col-0) and mutant (*skp2a*, *skp2b*, *and ceg*) seedlings were grown under control conditions (gray bar) or were treated with Sortin2 (black bar). The density of LRPs was evaluated after 72 hours. The mean and standard error of three experiments are shown (n = 30 [Col-0], 14 [*skp2a*], 13 [*skp2b*], and 12 [*ceg*] seedlings). The results were analyzed using Student's *t*-tests; brackets denote statistical differences between conditions (***$p < 0.001$; ns = not significant).

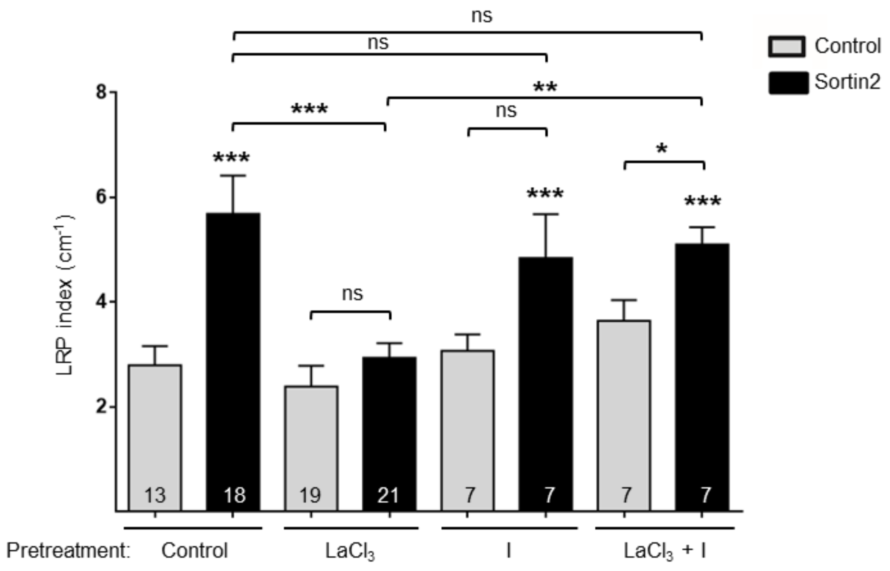

Supplementary Figure 9. LRP induction by endocytic trafficking requires early extracellular calcium ion entrance to the cell. Seven-day-old Col-0 seedlings were treated with $LaCl_3$ (1 mM), calcimycin (80 µM; I), or both $LaCl_3$ and calcimycin for 1 hour (pretreatment). Then, seedlings were either treated with Sortin2 (black bars) or grown under control conditions (grey bars) for 72 hours. LRPs were evaluated, and the results are shown as an index based on two experimental replicates. Numbers inside each bar indicate the number of scored seedlings in each condition. Results were analyzed using a one-way ANOVA with a Tukey's post-hoc test. Significant differences between the control condition (without Sortin2) and specific treatment are indicated above each bar; brackets denote statistical differences between conditions (*$p < 0.05$; **$p < 0.01$; ***$p < 0.001$; ns: not significant).

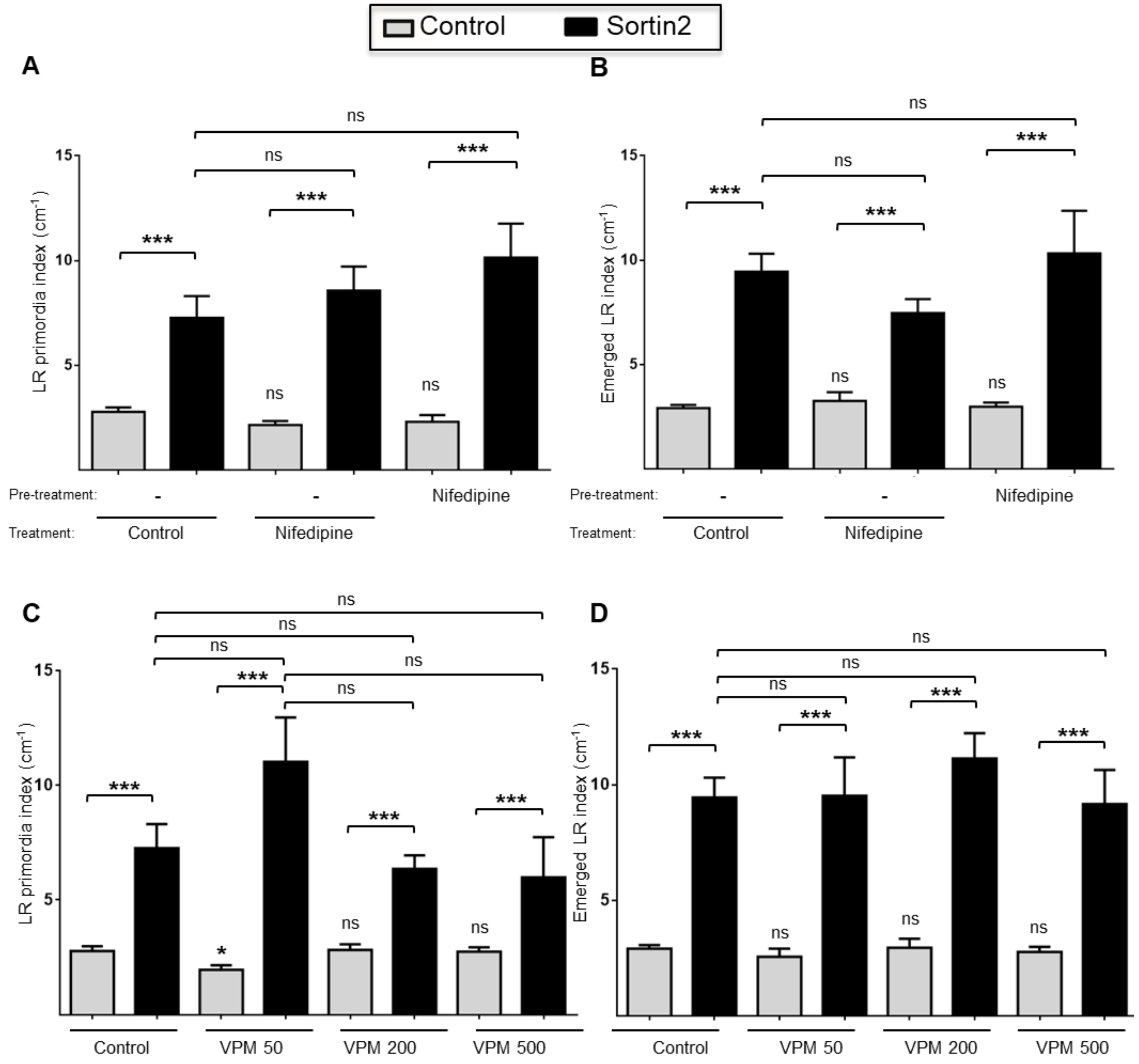

Supplementary Figure 10. Calcium channel inhibitors are unable to block Sortin2 LRP induction. Seven-day-old Col-0 seedlings were treated with the calcium channel inhibitors nifedipine (10 µM) (**A, B**) and verapamil (VPM; 50, 200, and 500 µM) (**C, D**) under Sortin2 treatment (black bars) or control conditions (grey bars) for 72 hours. A pretreatment with nifedipine (10 µM) for 60 minutes before Sortin2 exposure was also performed (**A, B**). After 72 hours of treatment, LRP (**A, C**) and emerged LR (**B, D**) indexes were quantified. Results from experimental replicates are shown (A and B, $n = 6$; C and D, $n = 10$ (Control), $n = 12$ (Sortin2), $n = 6$ (other conditions)). Significant differences between the control condition (without Sortin2) and specific treatments are indicated above each bar (one-way ANOVA with Tukey's post-hoc test); brackets denote significant differences between conditions (*$p < 0.05$; ***$p < 0.001$; ns: not significant).

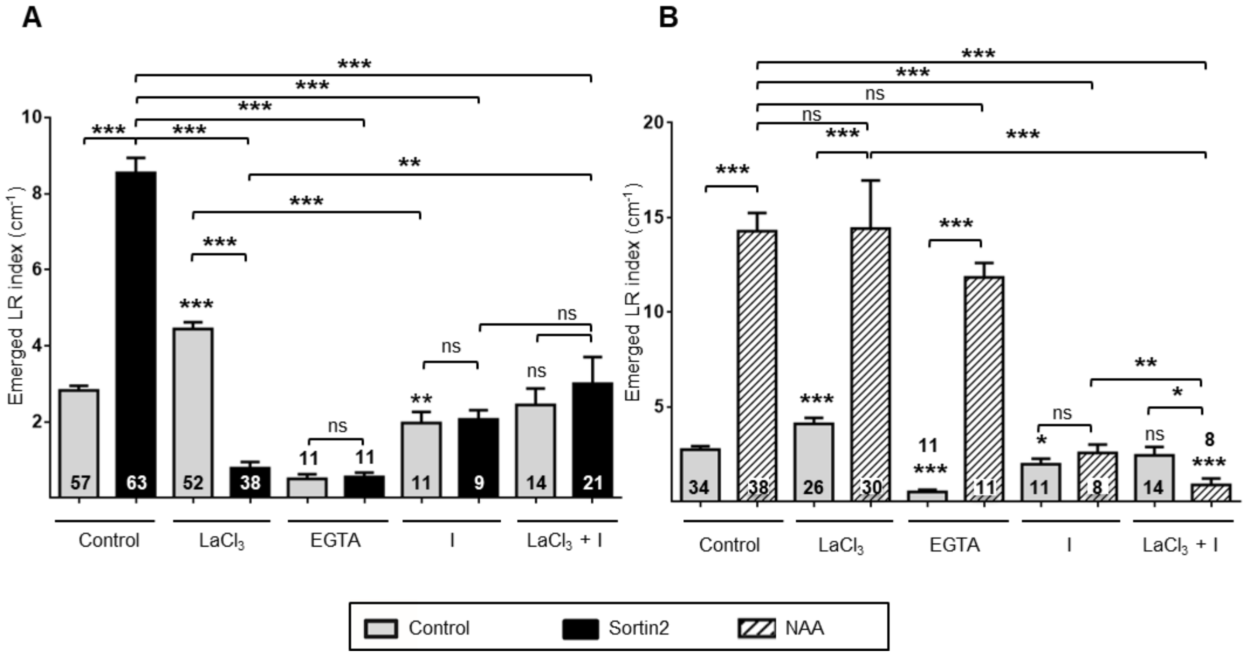

Supplementary Figure 11. Emerged lateral root induction by both Sortin2 and NAA is abolished by alteration of intracellular calcium level. Seven-day-old Col-0 seedlings were treated with Sortin2 (**A**) or NAA (1 µM) (**B**). Induction of LR emergence was analyzed in the presence of $LaCl_3$ (1 mM) and EGTA (10 mM) for 72 hours. Calcimycin (80 µM; I) was used to impair calcium gradients. Results from two experimental replicates are shown. Numbers inside each bar indicate the number of seedlings scored for each condition. Significant differences between the control condition (without Sortin2) and specific treatments are indicated above each bar (one-way ANOVA with Tukey's post-hoc test); brackets denote significant differences between conditions (*$p < 0.05$; **$p < 0.01$; ***$p < 0.001$; ns: not significant)
